# Can digitalization improve the equality and equity of food environment? Evidence from greengrocers in central Shanghai

**DOI:** 10.3389/fnut.2024.1452817

**Published:** 2024-11-05

**Authors:** Zhongyu He, Xiaoxu Chen

**Affiliations:** School of Architecture and Urban Planning, Nanjing University, Nanjing, Jiangsu, China

**Keywords:** digital food environment, environmental justice, same-day delivery, health equity, accessibility, online shopping

## Abstract

**Introduction:**

Online food shopping has a profound impact on people’s food acquisition behavior, the current study aims to understand how online food shopping may affect the accessibility of the local food environment and further influence the health equity among different populations.

**Methods:**

Taking 8512 traditional and online greengrocers in central Shanghai as an example, this paper uses Gini coefficient, location quotient and spatial clustering method to compare the equality and equity of food environment between physical and digital food outlets.

**Results:**

It finds that spatial equality is more significantly improved as a result of online food stores than are population equality and social equity of the food environment; older populations are not disadvantaged in terms of healthy food access but lower-income people are; the impact of online stores varies for different regions and different types of stores; depot-based stores have the most positive impact on health equity.

**Discussion:**

Policy implications are discussed to promote the environmental justice of healthy food accessibility.

## 1 Introduction

Diet is a major modifiable risk factor for disease morbidity and mortality (1). It has been widely acknowledged that local food systems have a direct impact on the population’s food decisions (2, 3). Increased availability of healthy foods will improve citizens’ diet nutrition and subsequently enhance their well-being and health (4, 5). However, a wealth of literature has suggested that neighborhoods with lower socioeconomic status suffer from poorer dietary habits (6, 7), partly due to their restricted access to healthy and affordable foods (8, 9). In recent years, digital technologies have permeated people’s everyday life globally and broadly reshaped the industry of food production, distribution and delivery (10). For example, data shows that by 2020, online food delivery services reached over 935 million users across the U.S., Europe, and China, with approximately 60% of these users accessing these services at least once a month (11). The outbreak of the Covid-19 pandemic further changed the way people acquire food and gave rise to a dramatic growth of online food shopping (12, 13). Among the various services to obtain food online, same-day delivery (SDD) is becoming prevalent owing to the proliferation of mobile shopping applications, especially in China; the SDD transactions for meals and fresh foods reached 461 billion and 195 billion Chinese Yuan, respectively, in 2018 (14). One feature of SDD that differentiates it from conventional online shopping is that orders are delivered from local stores or depots instantly (usually within an hour) (15); therefore, SDD is particularly favored for ordering of frequently purchased daily items such as prepared foods and groceries.

Although the digitalization of food environments, which means the use of digital technology to improve food production, distribution and acquisition of food information, is becoming a central issue in public health (16), little is known about whether and to what extent the digital food environment, in comparison with the physical food environment, impacts the accessibility to food resources, especially for the socioeconomically disadvantaged groups. By taking the greengrocers, namely food stores selling fresh fruits and vegetables, in central Shanghai as an example, the current study compares the accessibility of these food outlets between traditional physical stores and three types of SDD services, and further evaluates the influence of the digital food environment on the spatial equality, population equality and social equity for accessing healthy food at both city and subdistrict levels.

## 2 Literature review

### 2.1 Food environment and its digitalization

Food environment (or alternatively named nutrition environment) includes all places and pathways through which people acquire and/or consume food and the various characteristics of those environments that influence food choices (17). Scholars from various disciplines define food environment differently, which reflects its rich connotation and the problem-driven nature of its relevant research. The dimension of food environment can be divided into two domains: external and personal (18); the former refers to the availability and prices of food products, the characteristics of food vendors, and food marketing and regulation while the latter includes food accessibility, affordability, and desirability for consumers. Among these, accessibility is one of the major concerns for the fields of geography, transportation, and urban studies, and is also the focus of the current study. The accessibility of food environment is usually measured by the frequency (19), proximity (20) or presence (21) of food outlets. Using these techniques, the association between the accessibility of various types of food outlet and people’s health outcomes has been investigated (22–24); however, the results have been inconsistent for some types of food outlets (25). For example, although fast food restaurants are generally hypothesized to increase the consumption of unhealthy food and rates of obesity, which is confirmed by some studies (26, 27), other research report non-significant or reversed relationships (21, 28). As a result, instead of focusing on a specific type of food outlet, we choose greengrocers in our study as the research object, since fruits and vegetables may be the least controversial food options to contribute to a healthy diet.

The concept of digital food environment involves digital actors, digital settings, and digital activities performed by the digital actors in the digital sphere (29). The digital transformation of food environment has the potential to increase the accessibility of food options (30) and provide public health opportunities (31). In fact, digital and physical food environments are interconnected, rather than separate entities, and influence one another. For example, Xi et al. (14) argue that SDD online shopping substitute for local store shopping; Shi et al. (32) report that online shopping frequency is affected by the built environment via the mediating role of attitudes about shopping; He and Pan (13) find that physical and digital food outlets are highly integrated in Chinese communities, both of which significantly associate with food acquisition behavior. A recent scoping review in this field suggests that digital food environment research is more common in high-income countries and target at children and adults (16), which indicates a severe research gap in understanding the role of digitalization of food environments in low-and middle-income countries and for the elderly. In addition, there are different types of digital food outlets, whose accessibility has seldomly been examined separately in previous studies.

### 2.2 Environmental justice and health equity of food environment

Environmental justice is a major concern for urban policy makers; and two concepts, equality and equity, need to be established when “justice” is discussed. Although sometimes being used interchangeably, they are not synonymous with each other. While “equality” involves only a quantitative assessment, “equity” involves both a quantitative assessment and a subjective moral or ethical judgment (33). The notion of geographies of need by Harvey (34) suggests that localities with a larger presence of disadvantaged residents are in need for better access to public services and goods. Therefore, in our study we define “equality” as food resources distributed evenly across space or population and “equity” as food resources allocated in favor of disadvantaged social groups to meet their basic needs for healthy food.

Built environment has been increasingly identified as to associate with disparities in health behaviors and outcomes (35), and a wealth of literature has explored the inequitable distribution of health-promoting features of the built environment (e.g., parks and open green space) among low socioeconomic and racial and ethnic minority groups in environmental justice research (36–38). In recent years, planners and public health practitioners have recognized the importance of healthy food systems, as part of healthy built environments, in community planning and equity promotion (39). An area with restricted access to healthy and affordable foods is termed a “food desert” in literature (55); it is found to be more likely to appear in socioeconomically disadvantaged neighborhoods as measured by income level and ethnic composition (40, 41). However, such observations are less significant outside the North American context, where there is less pronounced residential segregation, especially with regard to ethnic backgrounds (42, 43).

Thus far, only a few studies have examined the impact of digitalization of food environment on the food access of disadvantaged social groups: Keeble et al. (44) find online access to prepared away-from-home food is higher in more deprived neighborhoods which may exacerbate the existing health inequity in England; a similar result is reported from a study in Chicago, Amsterdam, and Melbourne (45). Brandt et al. (30) find online grocery delivery services are rarely available in rural food desert census tracts in 8 states in the U.S. Sanchez-Diaz et al. (46) find online food delivery service is less accessible to disadvantaged and Covid-19 vulnerable populations in Sweden. These studies treat digital food environment as a separate entity and seldom consider its impact on the entire equity of food environment by making a comparison with physical food outlets, which we believe is a research gap and will be addressed in the current study.

## 3 Data and methods

### 3.1 Study area and data collection

**Figure 1:**
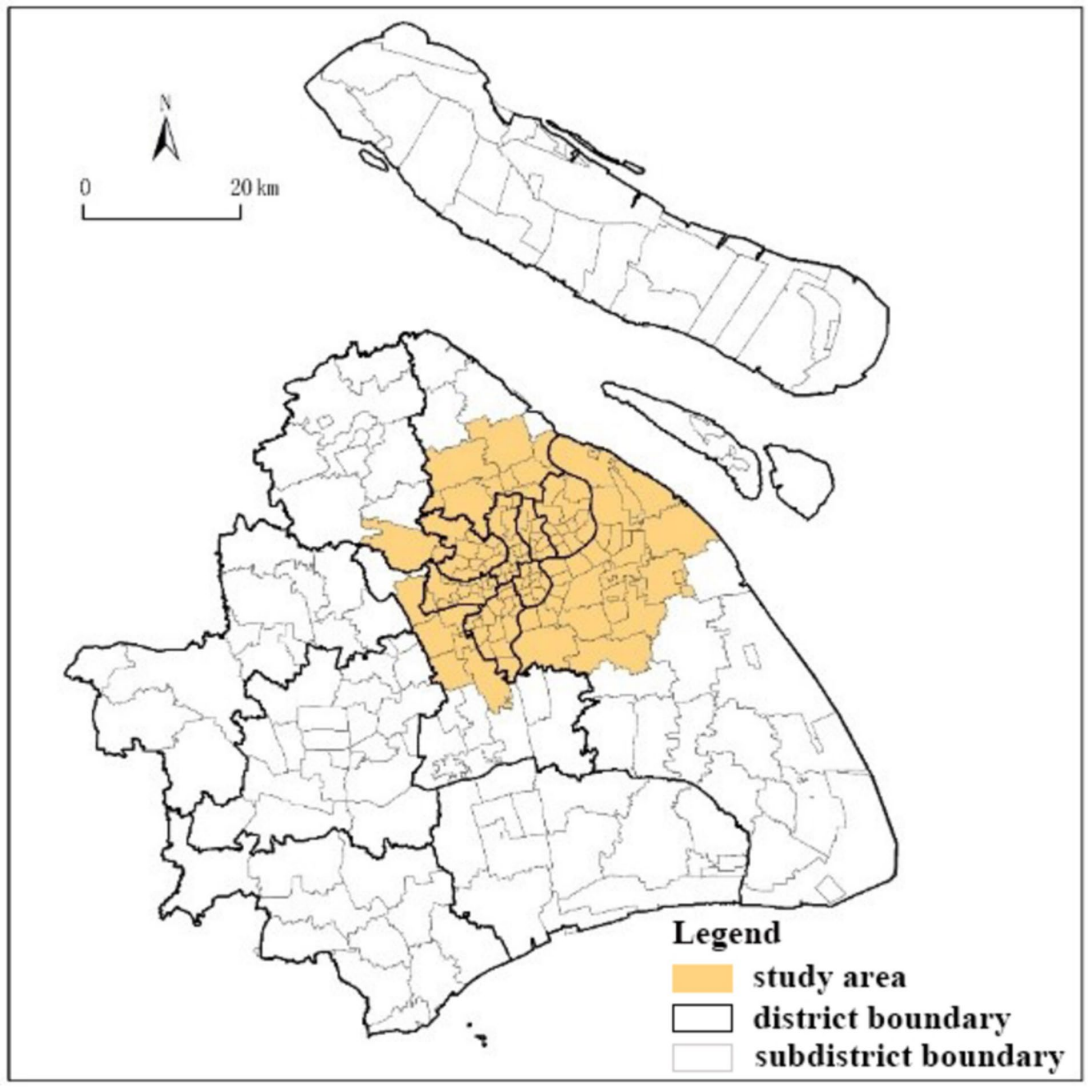
Location of study area in Shanghai.

Shanghai, with a population of approximately 25 million and a GDP *per capita* of about 26 thousand US dollars (47), is of the most developed online food shopping markets in China. The current study selected the central districts of Shanghai as the study area. The spatial structure of Shanghai is characterized by three concentric ring roads: the inner ring, the central ring, and the outer ring. According to the Shanghai’s master plan, the central city comprises the region within the outer ring road, with an area of 664 km^2^. The analysis unit in this paper is subdistrict (*Jiedao*), and there are 120 subdistricts in total located in or intersecting with the outer ring (Figure 1).

We divide the SDD service of fruits and vegetables in Shanghai into three types, according to its relation with traditional physical stores (Table 1). Type I stores mostly include small offline businesses accepting online orders by registering on an online marketplace that also provides the delivery service. Type II stores usually include larger market players that provide more variety of food choices and food delivery with their own logistic systems. Type III stores are depot-based and involve no physical stores.

**Table 1:** Definition of different physical and online stores.

Type	Business mode	Service coverage	Representative stores
Traditional store	Consumers need to visit and buy from a physical store personally.	1 km-radius circle (or 3.14 km^2^)	Supermarket, wet market, grocery store selling fruit or vegetable
Type I online store	Some of the traditional stores provide food delivery service. Consumers can either buy from the physical store or order via an online marketplace application.	3 km-radius circle (or 28.26 km^2^)	Meituan delivery
Type II online store	An integration of online platforms and offline stores. Consumers can either buy from a physical store or order online using the store’s application.		Hema Fresh, Yonghui Life, Jingdong 7fresh
Type III online store	Consumers order online and food will be delivered from a nearby depot. No physical store available.		Dingdong, Meituan Fresh

In existing literature, a threshold of 500 m was commonly used to assess food accessibility by walking (48). Considering residents in Chinese cities do grocery by motorcycling as well, we define the service coverage of traditional stores as a 1 km-radius circle. According to the delivery policy of many online stores, the maximum delivery distance for SDD is usually 3 km; therefore, the service coverage of the three online stores is defined as a 3 km-radius circle from the location of the store or depot.

We use point of interest (POI) data crawled from Baidu Maps^1^ to identify traditional stores selling fruits and vegetables and 5,502 such stores are found. For type I stores, we choose online greengrocers from Meituan, a SDD market leader with a market share of 68.2% according to a market survey by Trustdata^2^ and record 2,208 stores of this type. Type II and III stores are chosen based on the ranking of fresh food applications, and 584 and 218 stores are selected, respectively. In total, 8,512 stores are identified and geocoded using ESRI ArcMap 10.3. Other data used in this study includes housing price and population data. Housing price is crawled from Anjuke, a second-hand housing trading website.^3^ Population data is from WorldPop website^4^ at a 100 m grid; the population of the subdistrict is calculated by projecting and summing the population of each grid to the corresponding subdistrict.

### 3.2 Methods

#### 3.2.1 Measuring accessibility

We use the service area-based method (49) to calculate the accessibility of food outlets as Equation (1),


(1) $$\mathrm{Acces}s_{i}=M_{i}/A_{i}$$

where *Access_i_* is the accessibility of subdistrict *i*, *M_i_* is the sum of service area of all greengrocers (3.14 km^2^ for a traditional store and 28.26 km^2^ for an online store) in subdistrict *i,* and *A_i_* is the area of subdistrict *i*. The measurement considers the service coverage of stores outside a subdistrict if their service area intersects with the boundary of the subdistrict and includes those parts within the subdistrict.

#### 3.2.2 Measuring equality

Gini coefficient is a widely adopted statistical measure of economic inequality in a population and is used to measure the overall equality of the food environment in this paper. It is calculated as Equation (2),


(2) $$G=1-\overset{n}{\underset{k=1}{\sum }}\left(R_{k}+R_{k-1}\right)\left(P_{k}-P_{k-1}\right)$$

where *R_k_* is the accumulated proportion of accessibility in the *k*th subdistrict after ranking all the subdistricts from the least accessible food outlets to the most accessible as 1…*k…n*. *P_k_* is either the accumulated proportion of subdistricts or accumulated proportion of population in the *k*th subdistrict. When *k* = 1, the values of *R_k-1_* and *P_k-1_* are both 0. Gini coefficient ranges from 0 to 1, a Gini coefficient smaller than 0.2 indicates high equality while a Gini coefficient larger than 0.6 indicates extreme inequality.

Gini coefficient is an equality indicator of the whole study area; we use location quotient (50) to measure the local equality at the subdistrict level. Traditionally, it is used to measure a region’s industrial specialization relative to a larger geographic unit. In recent years it has been used to evaluate the concentration and equality of resources with spatial attributes (51). It is calculated as Equation (3),


(3) $$LQ_{i}=\left(M_{i}/P_{i}\right)/\left(M/P\right)$$

where *M* represents the sum of service area and *P* stands for the population. When a location quotient is larger than 1, it means the subdistrict has a higher concentration of food outlets than the average level of the study area.

#### 3.2.3 Measuring equity

In previous studies, social equity is usually measured using either regression model (9, 36) or spatial autocorrelation (37, 38). Here we use the latter, more specifically, a bivariate treatment of local indicator of spatial autocorrelation (LISA) (52) to identify patterns of association between food outlets accessibility and socioeconomic features of the subdistrict. It is calculated as Equation (4),


(4) $$I_{hk}=\left(X_{h}^{i}-\bar{X_{h}}\right)/\sigma_{h}\cdot \overset{n}{\underset{j=1}{\sum }}w_{ij}\cdot \left(X_{k}^{j}-\bar{X_{k}}\right)/\sigma_{k}$$

where *I_hk_* is the Moran’ I for food accessibility *h* and socioeconomic feature *k*; $\bar{X_{h}}$and $\bar{X_{k}}$ are the means of *h* and *k* while $\sigma_{h}$ and $\sigma_{k}$ are the variances of *h* and *k*; *i* and *j* denote subdistricts, and *w_ij_* is the spatial weight. Moran’s I value ranges from -1 to 1, 1 indicates a perfectly positive correlation between *h* of a subdistrict with *k* of the surrounding subdistricts while -1 indicates a perfectly negative correlation, and 0 suggests a random distribution without spatial clustering. We use two socioeconomic features: average housing price and proportion of older adult population (65 years and above) of the subdistrict as indicators of disadvantaged groups. As is discussed earlier, income is a widely used measurement for vulnerable populations; due to the unavailability of income data and the dominant proportion of real estate in Chinese families’ wealth, we use housing price to reflect the economic condition of residents. Ethnic segregation is usually not pronounced in most Chinese cities including Shanghai; we believe, however, that older adults deserve more attention in the discussion of health equity because of China’s rapid aging and the mobility barriers older adults face for food access. Therefore, we choose proportion of older adults as the second indicator to evaluate social equity.

## 4 Results

On the basis of the notion of “equality” and “equity” discussed in section 2.2, the following section explores food accessibility from three aspects: spatial equality, population equality, and social equity, which examines the distribution of food access across subdistricts, general population and disadvantaged population, respectively.

### 4.1 Spatial equality of the food environment

**Figure 2:**
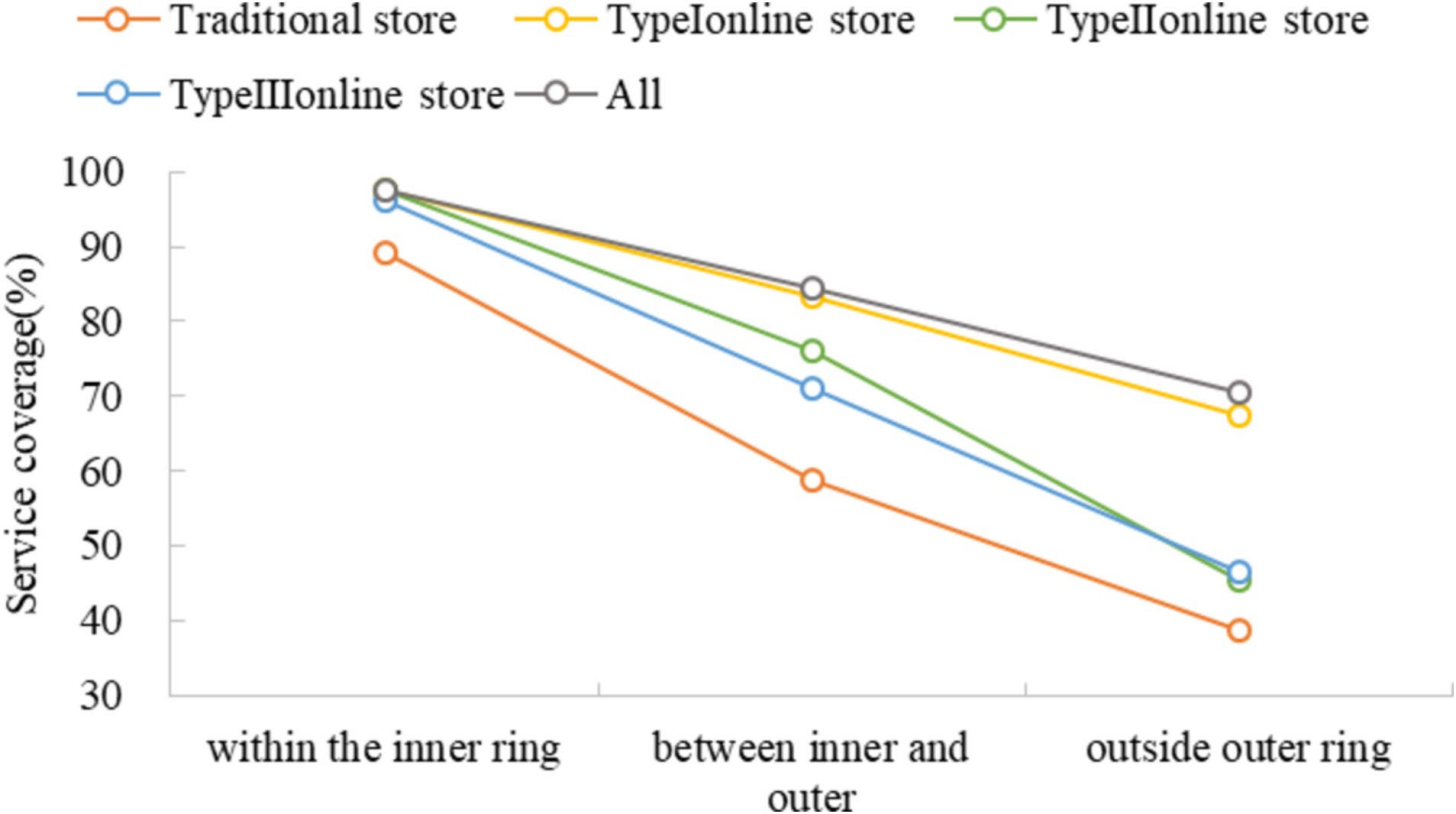
Service coverage of different stores stratified by regions.

**Figure 3:**
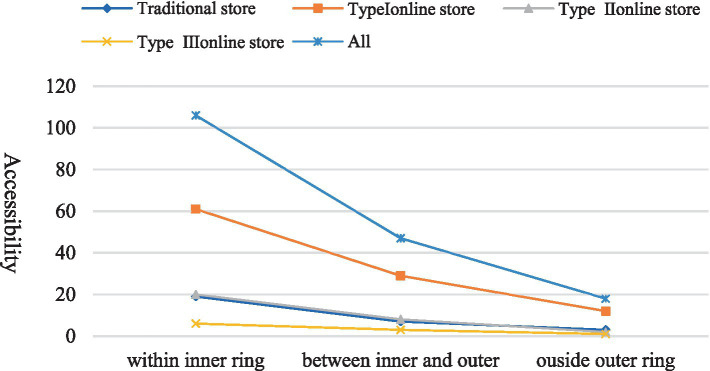
Food accessibility of different stores stratified by regions.

**Figure A1:**
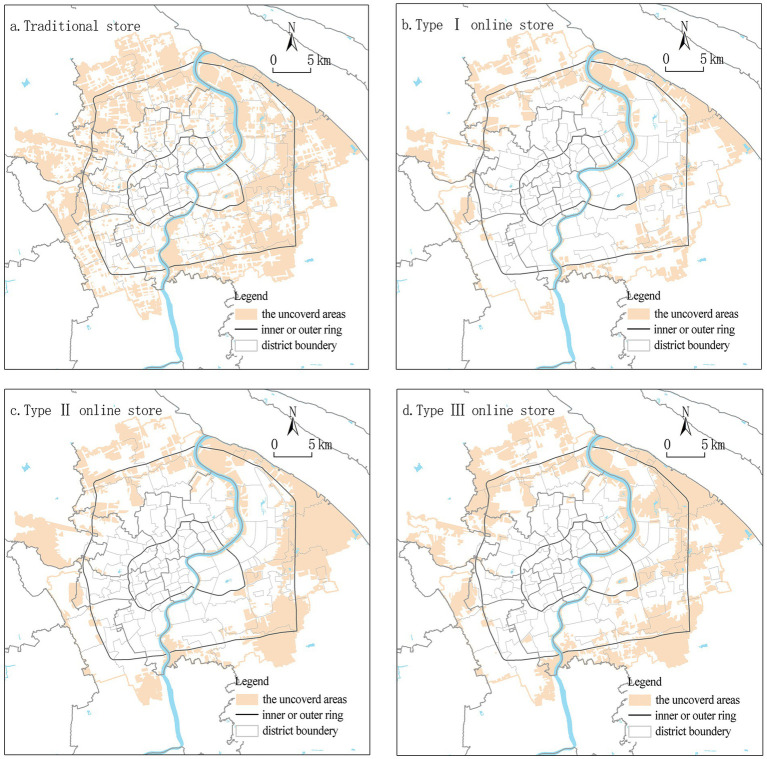
Service coverage of different types of greengrocers.

The service coverage of different types of greengrocers presents a common pattern declining from inter to outer ring roads (Figure A1). The number of traditional stores accounts for 65% of the total stores, but their service only covers 55% of the study area; Type I, Type II and Type III stores account for 26, 7 and 3% of the total stores, while their service covers 79, 68 and 65% of the study area, respectively. As a result, 81% of the study area is covered by any type of the stores. The difference in coverage between all the stores and the traditional store is 31, 25 and 9%, respectively, for regions outside the outer ring, between outer and inner ring, and within the inner ring (Figure 2). Further, we can see from Figure 2 that the supplementary coverage of Type I store is substantial for regions both outside the outer ring and between the outer and inner ring, while the supplementary coverage of Type II and III stores is more pronounced for regions between the outer and inner ring only. When we consider food accessibility, an opposite pattern is observed, where the supplementary effect is most substantial for the regions within the inner ring and least substantial outside the outer ring (Figure 3).

**Figure 4:**
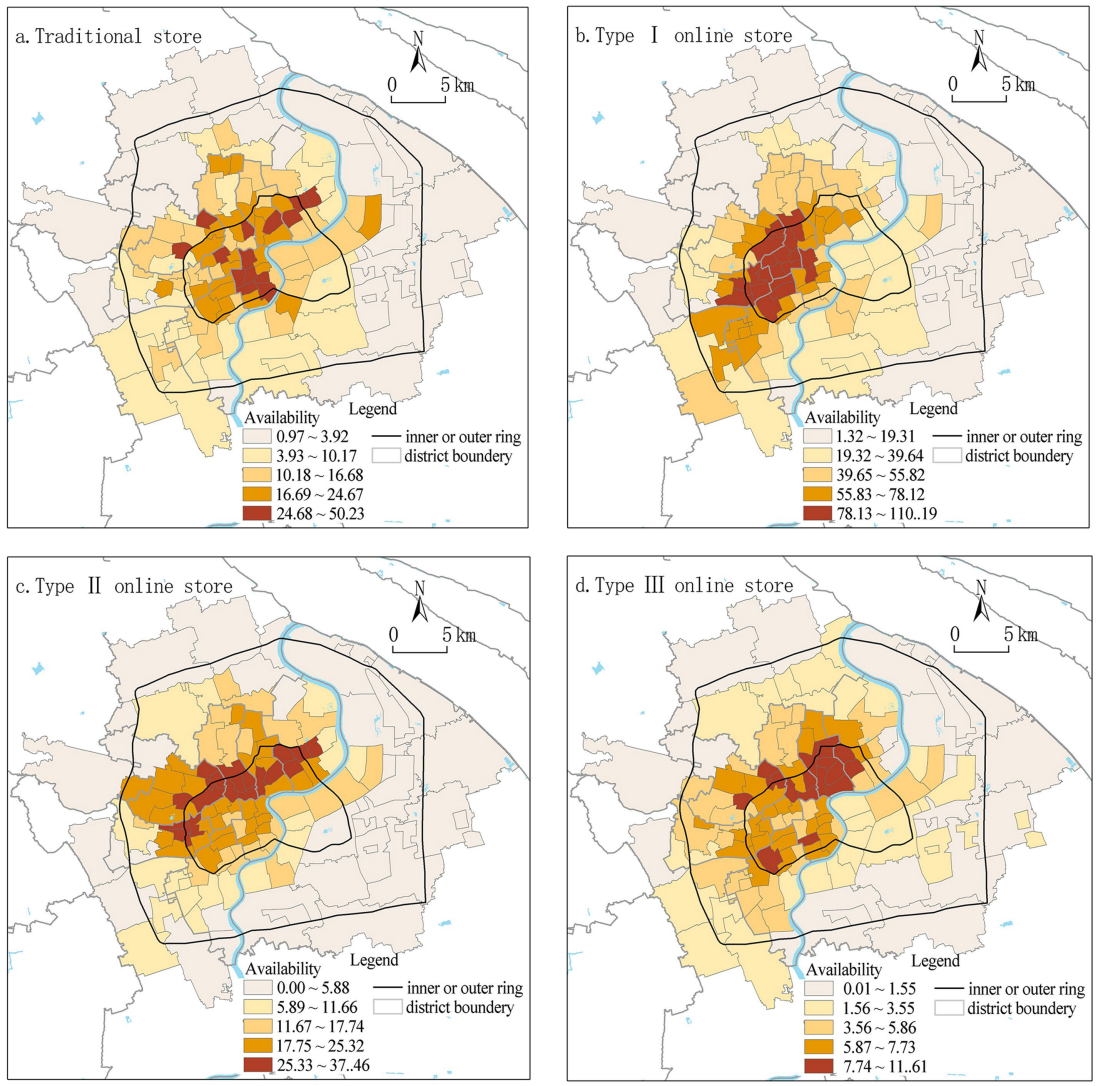
Food accessibility of different stores at subdistrict level Location quotient of different stores at subdistrict level.

Gini coefficient of food accessibility for different stores is Type III (0.329) < Type I (0.335) < Type II (0.358) < Traditional store (0.376), showing an acceptable level of equality. The overall Gini coefficient is 0.321, which indicates online stores improved the general spatial equality of the food environment. We use Jenks optimization method in ArcMap to explore food accessibility at subdistrict level (Figure 4) and we find that the accessibility of all types of stores declines as distance from city center increases; however, the location of different types of stores concentrates in different regions and therefore supplements each other in space. For example, subdistricts with highly accessible traditional stores are in the central part within the inner ring; subdistricts with highly accessible Type I stores are distributed in the southwest part within the inner ring and extend further to the southwest; subdistricts with highly accessible Type II stores are mainly distributed along the western and northern inner ring while subdistricts with highly accessible Type III stores are mostly located in the northeastern part of the inner ring.

### 4.2 Population equality of the food environment

Table 2 shows the statistics of population across 120 subdistricts. Gini coefficient of food accessibility based on population for different stores is Type III (0.427) < Type II (0.456) < Type I (0.458) < Traditional store (0.460). The overall Gini coefficient is 0.445. Online stores again improve the general equality of food accessibility, although the population dimension is less equal compared with the spatial dimension.

**Table 2:** Socioeconomic features of the subdistrict.

	Maximum	Minimum	Mean	S.D.
Population (thousand)	406	30	117	73
Prop. of older adults (%)	35	18	27	4
Average housing price (yuan/m^2^)	133,833	25,443	61,758	18,634

**Figure 5:**
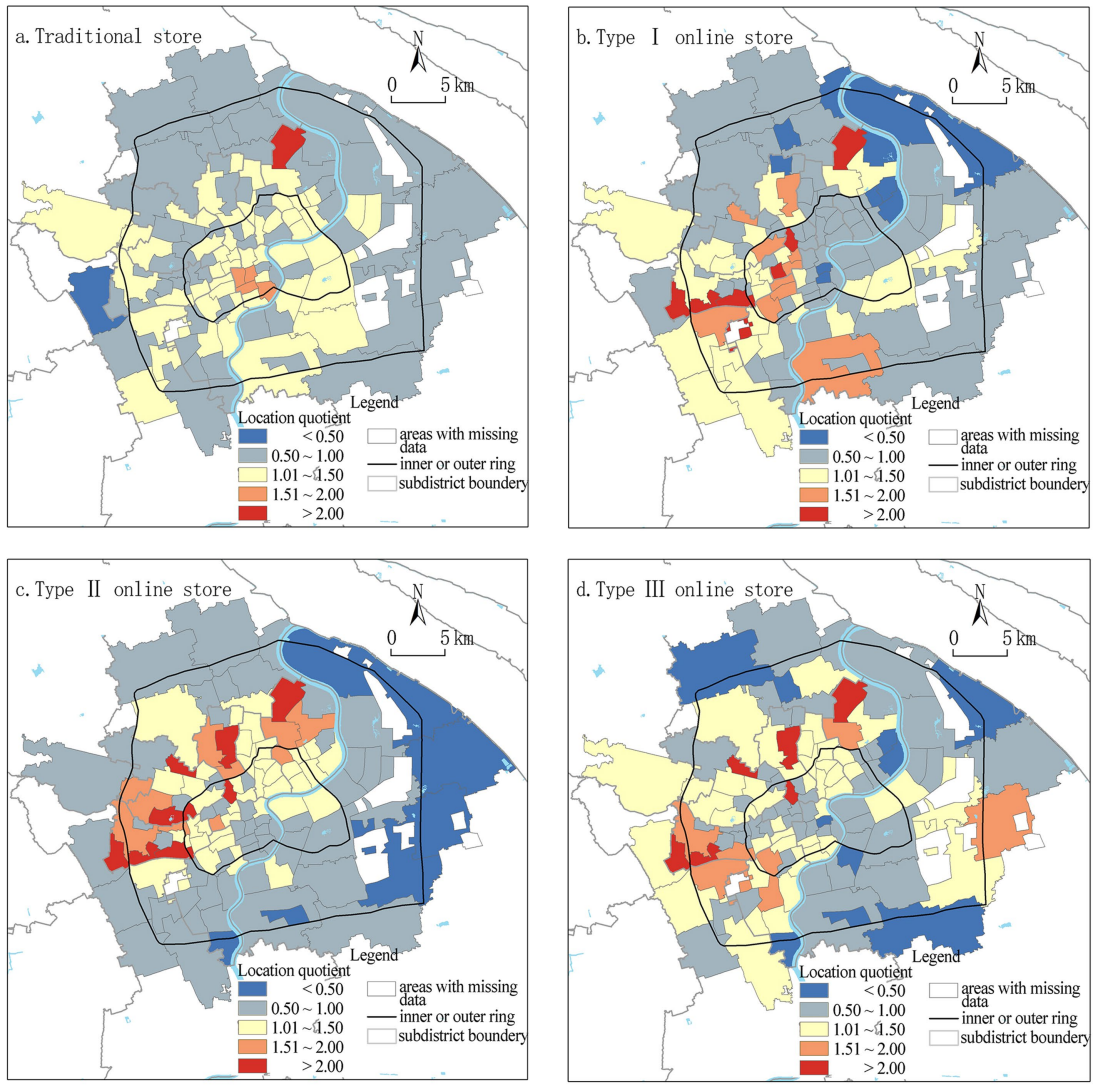
Location quotient of different stores at subdistrict level.

Further, the location quotient of each subdistrict is calculated and divided into five categories (<1/2 times mean, 1/2 ~ 1 times mean, 1 ~ 1.5 times mean, 1.5 ~ 2 times mean, and above 2 times mean). The spatial pattern of location quotient is more complicated (Figure 5): compare with three types of SDD stores, the distribution of traditional store and population is more balanced, with fewer subdistricts having extreme high or low location quotients. Subdistricts with high location quotients for Type II and Type III stores are mainly in the western and northern parts between the inner and outer rings. Subdistricts with very low location quotients for all types of stores are in the periphery of the central city. The result indicates when population is controlled, the distribution of online food outlets is more concentrated than traditional stores, namely regions between the inner and outer rings have a higher concentration of accessible online greengrocers *per capita*.

### 4.3 Social equity of the food environment

#### 4.3.1 Spatial correlation between food accessibility and proportion of older adults

Pearson correlation analysis shows that accessibility of four types of greengrocers are highly correlated with each other, and at the same time all highly correlated with proportion of older adults (Table 3). Online stores have a higher correlation with older adults than traditional stores, which indicates digital food outlets may have a positive role in improving the social equity of food accessibility.

**Table 3:** Pearson correlation of food accessibility and socioeconomic features.

	Traditional store	Type I store	Type II store	Type III store
Traditional store	1	0.685^**^	0.774^**^	0.758^**^
Prop. of older adults	0.627^**^	0.695^**^	0.652^**^	0.712^**^
Average housing price	0.513^**^	0.644^**^	0.384^**^	0.442^**^

** significant at 0.01 level.

**Figure 6:**
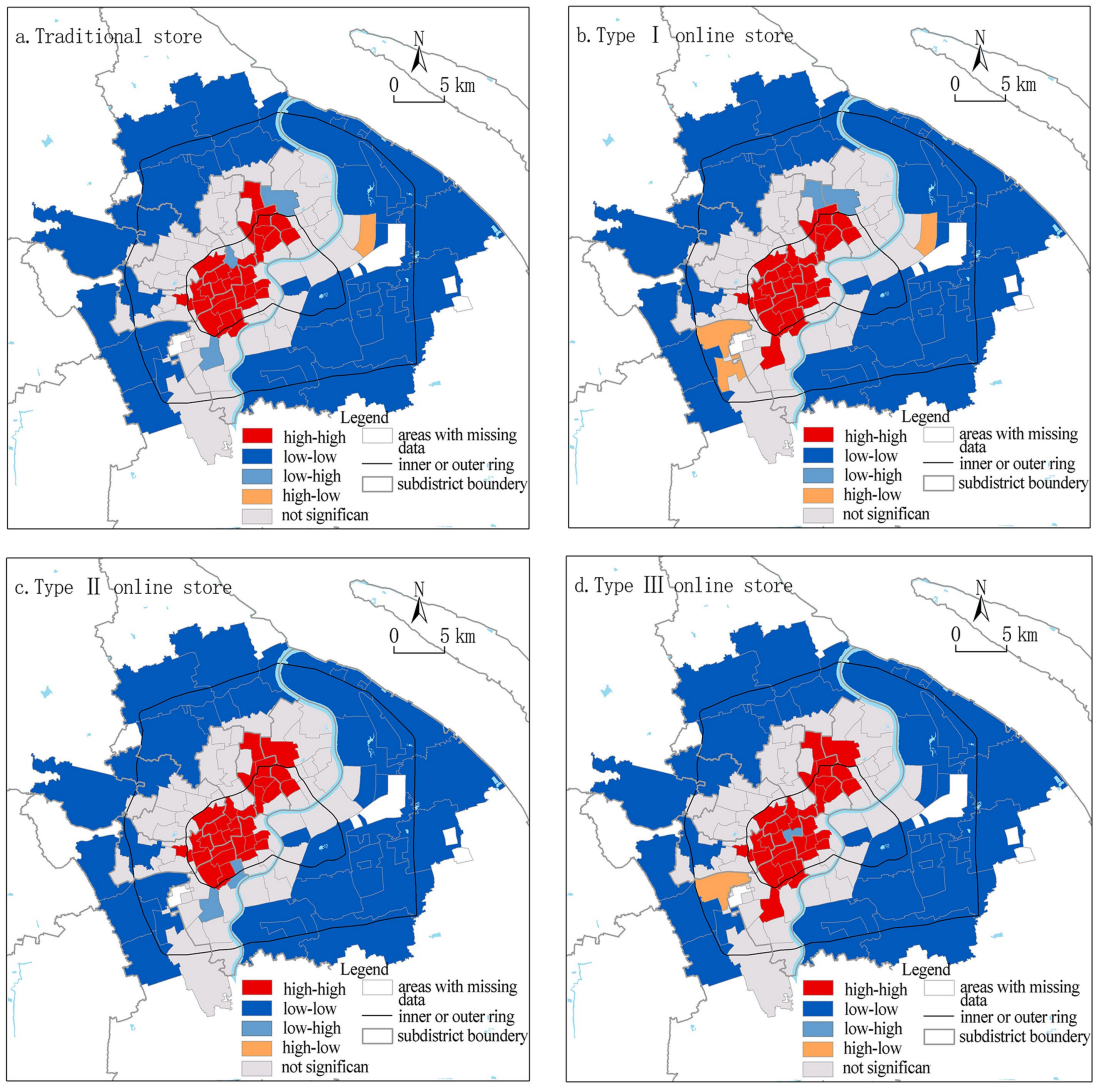
Bivariate LISA results for food accessibility and proportion of older adults.

Bivariate LISA cluster (Figure 6) further presents the spatial correlation between food accessibility and proportion of older adults at the subdistrict level. Traditional and online stores show a similar pattern of clustering, with the west side of the Huangpu River within the inner ring presenting a High-High cluster, the periphery of the study area presents a Low-Low cluster, and the subdistricts between the inner and outer rings show random distributions. The limited number of Low-High clustering (low food accessibility with high proportion of older adults) suggests that regarding aging population, the food environment is fairly equitable in Shanghai; and three subdistricts of this pattern for traditional stores switch to High-High pattern for online stores, which suggests the equity is improved with the digitalization of food environment.

#### 4.3.2 Spatial correlation between food accessibility and housing price

**Figure A2:**
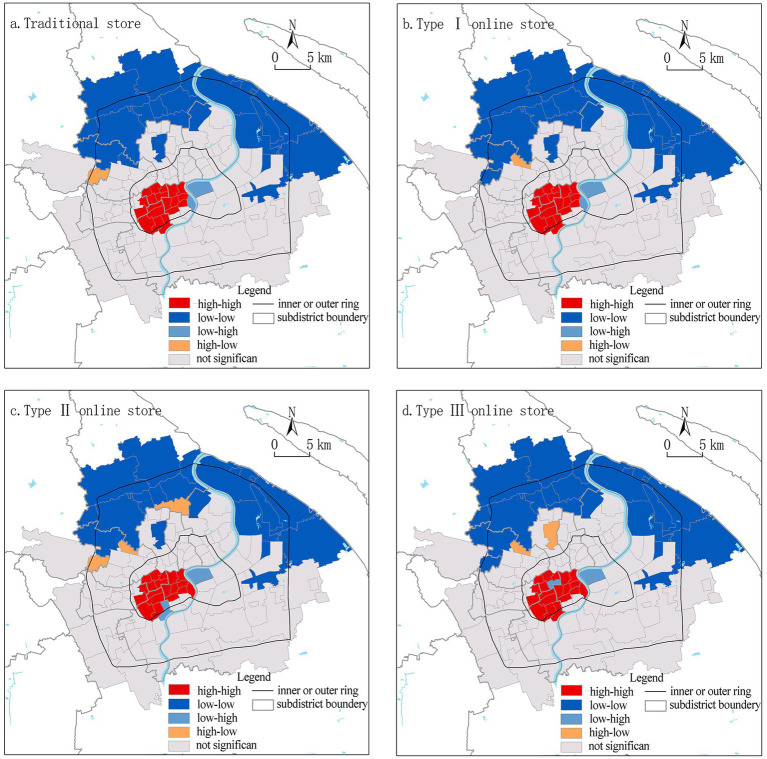
Bivariate LISA results for food accessibility and average housing price.

Bivariate LISA cluster for food accessibility and housing price at subdistrict level (Figure A2) again presents a similar pattern between four types of stores. However, subdistricts with significant clustering are dramatically reduced compared with the correlation between food accessibility and older population. High-High clustering concentrates in the southwest subdistricts within the inner ring, which mostly are the gentrified region of Xuhui and Huangpu Districts. Low-Low clustering concentrates along the northern outer ring. The only subdistrict with a constant Low-High cluster (low food accessibility and high housing price) is Lujiazui-the Central Business District of the city, where upscale apartments and office towers concentrate with fewer greengrocers. The number of subdistricts with High-Low cluster (high food accessibility and low housing price) increases from 1 for traditional stores to 4 for online stores, which is an improvement for social equity of the food environment.

## 5 Discussion

### 5.1 Equality and equity of food access for traditional food stores

Improving access to food outlets of fresh fruits and vegetables for disadvantaged groups may promote a healthy diet and contribute to the health equity among populations. Although many studies have noted the inequity of healthy food accessibility among urban residents (6, 41) and the strengthening residential segregation in China (53), our empirical study reports a mixed result: at the city level, the spatial equality of physical food outlets is reasonable with a Gini coefficient of 0.376 but the population equality needs improving with a Gini coefficient of 0.460; at the subdistrict level, the accessibility declines from city center to outer ring road, which has a similar distribution pattern as the population density. We consider two indicators of a subdistrict’s socioeconomic status: proportion of older adults and average housing price. According to the findings, with regard to accessing greengrocers in central Shanghai, older people living in most subdistricts are not disadvantaged; however, low-income residents living in the northern part of the central city have lower access to these stores. We argue this result is partly due to the overlap of wealthy residents, older population (mostly native residents), and concentration of retailing in the inner city. To a certain extent, it is a coincidence rather than a planned outcome. This result is consistent with an earlier study in Hangzhou (7) where healthy food access was found to be positively associated with some socioeconomic indicators (e.g., proportion of the less educated, unemployed, children) while negatively associated with some others (e.g., proportion of illiterate) at subdistrict level. Another study concerning the social equity of green space access in Shanghai (38) found that low-income social groups were not disadvantaged in terms of access to urban parks and claimed that planning regulations can balance out some of the institutional and market inequalities. In fact, traditional markets in Chinese cities are planned and built by the government based on population density; and recently, the municipal government of Shanghai has been making efforts to promote building “15-min pedestrian scale neighborhoods,” which aim to guarantee a walkable distance to all daily community facilities. These planning strategies contribute to less inequity of physical food environment in Shanghai compared with Western countries. Some low-income residents living in periphery regions of the central city, however, may not have enough access to healthy food and therefore deserve more attention.

### 5.2 Impact of digital food stores on equality and equity of food access

The distribution of three types of online food stores is highly correlated with traditional stores (Pearson coefficients: 0.685–0.774, *p*-value<0.01), which indicates physical and digital food environments in Chinese cities are well integrated (13). Although the service of online greengrocers does not evenly cover the population and different social groups, as previous studies suggested (30, 46), we find they exert a positive impact on the equality and equity of the whole food environment. Such an impact is more significant in terms of spatial equality (i.e., service coverage and food accessibility), but less significant regarding population equality and social equity. For population equality, the three types of online stores all show smaller Gini coefficients than traditional stores; however, the location quotient analysis implies that the distribution of online stores presents more extreme situations (LQ > 2 or < 0.5). Some subdistricts between the inner and outer rings have a location quotient lower than 1 for traditional stores but a location quotient higher than 1.5 or 2 for online stores; on the other hand, some subdistricts with extremely low location quotient for online stores have a higher location quotient for traditional stores. Therefore, physical and digital food outlets supplement each other (14) in these regions and as a result the overall equality is improved.

The impact also varies for different regions and different types of stores: regions outside the outer ring benefit most for service coverage, and regions within the inner ring benefit most from food accessibility while regions between the outer and inner rings benefit most from population equality; Type III stores (depot-based) have the most balanced distribution across subdistricts and population, and also have the most positive impact on social equity of the healthy food access. Type I stores show the least significant role in terms of improving social equity. We argue this is due to the location choice of depots follows a different mechanism (location with lower rent but less exposed to customers) with other actual stores (54) and therefore has a more balanced coverage and provides a more equitable access to disadvantaged groups.

### 5.3 Limitation of the current study and implication for future studies

One limitation of the current study is the scale of the analysis unit is relatively large due to data availability. Previous studies show that the association between access to healthy food and socioeconomic status may differ between subdistrict and census tract (9). A smaller analysis unit can better reflect the heterogeneity of socioeconomic status of neighborhoods and enable a more precise evaluation of food access equality and equity. Another limitation is our definition of accessibility may be subject to possible Modifiable Areal Unit Problem (MAUP). In this study, we chose 1 km as the boundary of the service area for traditional stores; although this distance is among the scope of most commonly used buffer sizes to measure community food environment (25), previous studies have shown that the threshold of the buffer can have an impact on the result. To minimize this problem, sensitivity analysis can be carried out to test the effect of various buffer sizes.

While this study presents a snapshot of the current state of digital food environment, a longitudinal perspective will add significant value of how digitalization’s impact on equality and equity changes over time. Future research could incorporate this approach to build on the current findings. In addition, securing equitable access to healthy food for vulnerable groups may not be sufficient to guarantee a change of dietary choices (22, 42); other factors such as price, consumer perception and preference should be considered. Future study could also incorporate more data on consumer experiences and food acquisition behavior to understand how different demographics navigate these digitalized food environments, which will provide a more holistic view of the equity implications.

## 6 Conclusion

By comparing the traditional greengrocers with three types of SDD stores in central Shanghai, this paper reveals the following findings: first, food environment in the study area is barely satisfactory in terms of equitable access to healthy food, which is due to the interplay of market forces, planning effort and long-developed urban structure; second, online food shopping generally improves the food-environmental justice, but the impact is more significant for spatial equality and less for population equality and social equity, lower-income residents in the urban periphery may suffer from insufficient access to healthy food; third, the benefits of digitalization of food environment vary across regions and store types, and depot-based online stores have the most positive impact on health equity.

There is a long-standing debate in China as to whether the platform economy should be encouraged due to concerns that it will damage the non-digital economy. However, with regard to the food industry, the importance of online food shopping has been widely acknowledged since the Covid-19 outbreak due to the frequent lockdown of communities. In this paper, we further find that the digital food environment has the potential to improve the equality of food accessibility as well as the health equity for socioeconomically disadvantaged groups. Therefore, the digitalization of physical food stores should be encouraged, as it will further strengthen the integration and interaction between physical and digital food environments. Meanwhile, depot-based online stores should receive more attention in the planning of “15-min pedestrian scale neighborhoods” as they are shown to have the most significant impact on social equity to access healthy food. In addition, for regions identified as “food deserts” (such as the northern subdistricts along the outer ring in this paper), local governments can encourage nearby online food stores to extend their delivery distance by subsidizing either the stores or the consumers. It is also a crucial issue for policymakers to realize that technological and infrastructural barriers may prevent equitable access. For example, in this paper, the technical barrier of using mobile apps may discourage older adults buying fruits and vegetables online even when their neighborhood is covered by the delivery service. Therefore, together with planning interventions, other actions such as to improve internet access, digital literacy and lower the cost associated with digital tools should also be considered to positively affect different stakeholders within the food environment.

## Data Availability

The raw data supporting the conclusions of this article will be made available by the authors, without undue reservation.

## Footnotes

^1^ https://map.baidu.com

^2^ http://www.itrustdata.cn/

^3^ https://anjuke.com

^4^ https://www.worldpop.org
